# Adaptive optics imaging of inherited retinal diseases

**DOI:** 10.1136/bjophthalmol-2017-311328

**Published:** 2017-11-15

**Authors:** Michalis Georgiou, Angelos Kalitzeos, Emily J Patterson, Alfredo Dubra, Joseph Carroll, Michel Michaelides

**Affiliations:** 1 UCL Institute of Ophthalmology, University College London, London, UK; 2 Moorfields Eye Hospital NHS Foundation Trust, London, UK; 3 Department of Ophthalmology and Visual Sciences, Medical College of Wisconsin, Milwaukee, Wisconsin, USA; 4 Department of Ophthalmology, Stanford University, Palo Alto, California, USA

**Keywords:** vision, retina, imaging, genetics

## Abstract

Adaptive optics (AO) ophthalmoscopy allows for non-invasive retinal phenotyping on a microscopic scale, thereby helping to improve our understanding of retinal diseases. An increasing number of natural history studies and ongoing/planned interventional clinical trials exploit AO ophthalmoscopy both for participant selection, stratification and monitoring treatment safety and efficacy. In this review, we briefly discuss the evolution of AO ophthalmoscopy, recent developments and its application to a broad range of inherited retinal diseases, including Stargardt disease, retinitis pigmentosa and achromatopsia. Finally, we describe the impact of this *in vivo* microscopic imaging on our understanding of disease pathogenesis, clinical trial design and outcome metrics, while recognising the limitation of the small cohorts reported to date.

## Introduction

Inherited retinal disease (IRD) is the leading cause of legal blindness in England and Wales among the working age population and the second most common in childhood.^1^ IRD is a group of clinically heterogeneous conditions, which can result in diagnostic challenges, often thereby necessitating detailed multimodal retinal imaging, as well as electrophysiological and psychophysical evaluation. They are subject to a broad range of research avenues and interventions which have been recently reviewed.^2^ Here, we categorise IRDs on the basis of natural history (stationary or progressive) and the primarily affected retinal cell type.

*In vivo* retinal imaging has been rapidly evolving over the last decades primarily due to advances in optics, electronics and computer technology. The introduction of optical coherence tomography (OCT) has revolutionised the clinical investigation of retinal diseases.^3 4^ One of the main limiting factors for *in vivo* retinal imaging is ocular aberrations, due to the optical imperfections of the eye.^5^ Adaptive optics (AO) can be employed in ophthalmology to overcome the aforementioned limitation.^6^

## Brief overview of AO retinal imaging

The incorporation of AO to any ophthalmoscopic technique, including fundus photography, OCT and scanning laser ophthalmoscopy (SLO), provides *in vivo* microscopic imaging.^6–9^ AO ophthalmoscopes typically use a wavefront sensor to measure the ocular monochromatic aberrations and a deformable mirror to correct for the detected aberrations.^6 9 10^ Herein we will be focusing on AOSLO photoreceptor imaging as this is the modality that has been most extensively used in patients with IRD. By focusing a scanning light source on the photoreceptor layer and rejecting out-of-focus light through the use of a confocal aperture, axial sectioning is achieved, thereby increasing image contrast.^8 11^ Photoreceptors with relatively intact outer segments waveguide some incident light, and backscatter a very small fraction (less than 0.1%), which is used for imaging.^12^ When collecting that light in a confocal detector, the cone^8 13 14^ and perifoveal rod^8 15 16^ mosaics can be resolved. Several systems have been developed including both custom-built and commercially available devices.

The non-confocal backscattered light can also be exploited to reveal the photoreceptor inner segment mosaic. For example, the split detection (SD) technique (SD-AOSLO) does so by subtracting images created by capturing the light to the left of the confocal aperture with one detector and the light to the right of it with a different one.^17^ This recent development was transformational because cones with compromised outer segments (as would be anticipated in the majority of IRDs) can now be reliably identified for the first time. This has major implications for patient stratification and targeting of intervention.^18–25^

**Figure 1:**
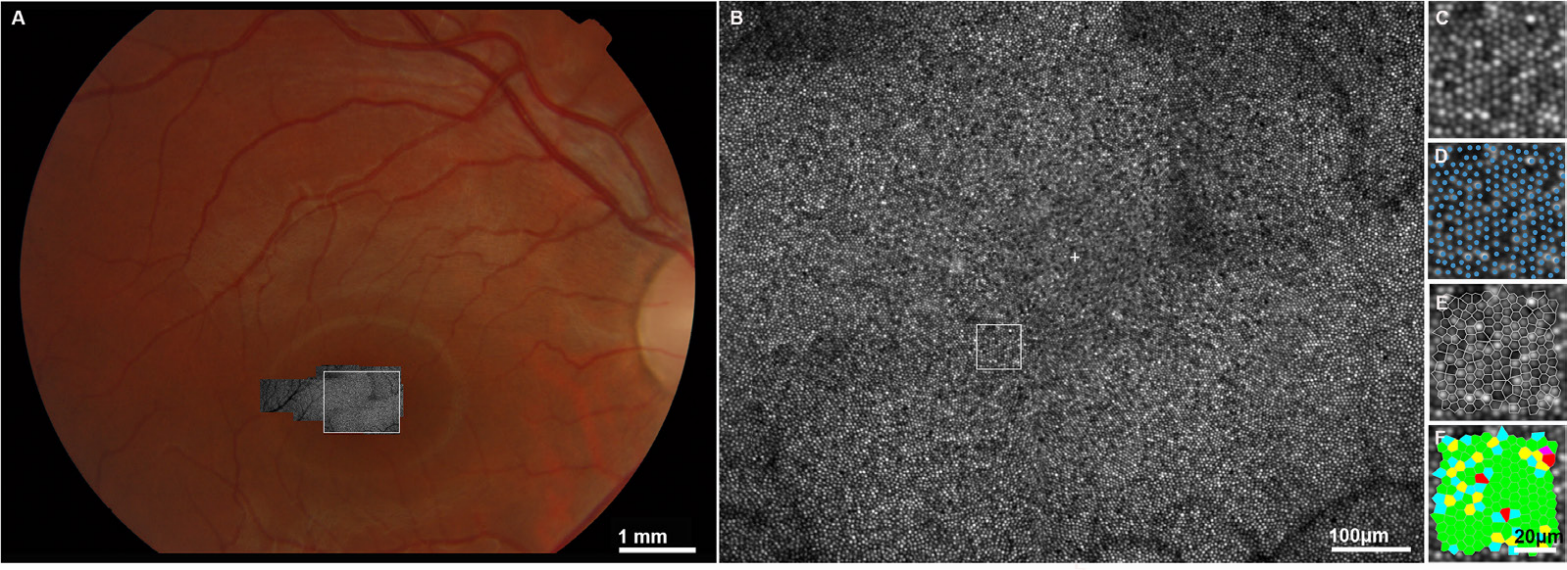
Adaptive optics scanning laser ophthalmoscopy (AOSLO) imaging of a healthy subject and cone quantification. (A) Colour fundus photograph (30°) of a healthy subject (MM_0136), with AOSLO montage superimposed. The white square encompasses the foveal avascular zone (region of interest, ROI), which is magnified in (B). (B) Confocal AOSLO of the ROI, the estimated foveal centre is marked with a white cross and the 55 μm×55 μm area of sampling for cone counting with a white box at 0.35° from the foveal centre. Scale bar=100 μm. (C) Magnified view of the sampled area. (D) The sampled area with cones marked. (E) The sampled area with Voronoi domains. (F) The Voronoi representation coloured according to the number of neighbouring cells. Green represents six-sided bound cones. Scale bar for (C–F)=20 μm.

Due to light safety restrictions, each individual AOSLO raw frame is captured using very low illumination power (~100 µW at the pupil) and thus the resulting images are inherently noisy. Therefore, AOSLO image sequences are captured at each retinal location of interest, and used to create a higher signal-to-noise ratio (SNR) image by averaging a few of these frames after correcting for eye motion and scanning distortions.^26^ These high SNR images are then stitched together to create a larger montage (figure 1). A range of photoreceptor metrics have been employed to date, with cone density for a given eccentricity being the most widely used, and usually compared with normative data from histology^27^ or imaging studies.^13 28 29^ Other metrics include (1) cone spacing—average distance between cells in a given location, (2) Voronoi analysis (figure 1) which involves counting the number of neighbouring cells based on the distance between them, thereby assessing mosaic geometry,^28^ (3) reflectivity,^18^ and (4) metrics for the preferred orientation of cones and local spatial anisotropy.^30^

The combination of OCT and AO (AO-OCT) is an evolving field, aiming for 3D reconstruction and offers greater axial resolution compared with AOSLO.^31^

## IRDs and AO retinal ophthalmoscopy

The selected conditions below have been prioritised based on the ability of published AO ophthalmoscopy studies to demonstrate clinical, research or trial utility. There are inherent limitations due to the often small cohorts reported to date. These are usually small due to the vast genetic and phenotypic heterogeneity of IRDs, the low prevalence of each genotype and due to the difficulty of establishing multicentre studies given the limited availability of AOSLO. However, similar limitations are often faced by other studies using other modes of high-resolution imaging. For clarity, we have included the number of subjects in each study we describe and whether the patients were molecularly confirmed (online supplementary table 1).

10.1136/bjophthalmol-2017-311328.supp1 Supplementary file 1

### Macular dystrophies

#### Stargardt disease

Stargardt disease (STGD1) is the most common form of hereditary macular dystrophy.^32^ Confocal AOSLO (cAOSLO) has demonstrated abnormal and decreased cone spacing in regions corresponding to areas of reduced and irregular fundus autofluorescence (FAF), in predominantly late-onset/foveal sparing molecularly proven patients (11 of 12 patients).^33^ Moreover, foveal retinal pigment epithelium (RPE) cells were imaged in areas where the photoreceptor mosaic appeared disrupted in confocal reflectance imaging, suggesting photoreceptor loss preceding RPE cell loss—although the application of SD-AOSLO would address whether there are in fact cone inner segments present.^33^ Song *et al* also reported increased photoreceptor spacing, in genetically proven STGD1 (n=2), in otherwise normal appearing areas on OCT and FAF imaging; also consistent with photoreceptor loss preceding clinically detectable RPE disease.^34^ Interestingly, SD-AOSLO derived cone density has been shown to correlate well with OCT measurements of outer nuclear layer thickness and retinal sensitivity (n=14; all molecularly confirmed), demonstrating a valuable structure-function association, even though the extent of atrophic changes was not corresponding to visual aquity.^19^ Using cAOSLO and SD-AOSLO, Tanna *et al* investigated the reliability and repeatability of cone counting in patients with STGD1 (n=12), suggesting superior reliability and repeatability with SD-AOSLO.^35^

Longitudinal imaging studies of the photoreceptor and RPE mosaic in large molecularly proven specific STGD1 cohorts (ie, childhood-onset, adult-onset and late-onset/foveal sparing) are needed to evaluate cellular disease progression and potentially identify the most suitable participants for ongoing and multiple planned gene therapy and pharmacological interventions.^19 33 34 36^ AO ophthalmoscopy may be a useful method of monitoring in trials, since ‘classic’ parameters of ophthalmological examination including best corrected visual acuity (BCVA) are not sufficiently sensitive outcome measures for conditions such as STGD1.^37^

#### Best disease

Normal photoreceptor structure and cone densities in areas adjacent to clinically visible lesions have been reported, with persistent photoreceptor structure overlying stage 1 and 2 vitelliform Best disease lesions, in keeping with relatively intact visual acuity (VA).^38^ Using cAOSLO and SD-AOSLO, variable photoreceptor architecture has been observed to be associated with different stages of the disease and the location within the lesions, including reduced cone density, due to major discontinuities/gaps in the mosaic, and cone inner segment enlargement.^23^

#### X-linked retinoschisis

Duncan *et al* 39 have reported increased and irregular cone spacing within the foveal schisis characterising X-linked retinoschisis. Interestingly, cone spacing was normal and regular elsewhere. The preserved waveguiding cones at the fovea and eccentric macular regions may indicate increased likelihood of successful rescue with intervention—and could also be helpful in patient selection.

### Stationary dysfunction syndromes

#### Cone dysfunction syndromes

**Figure 2:**
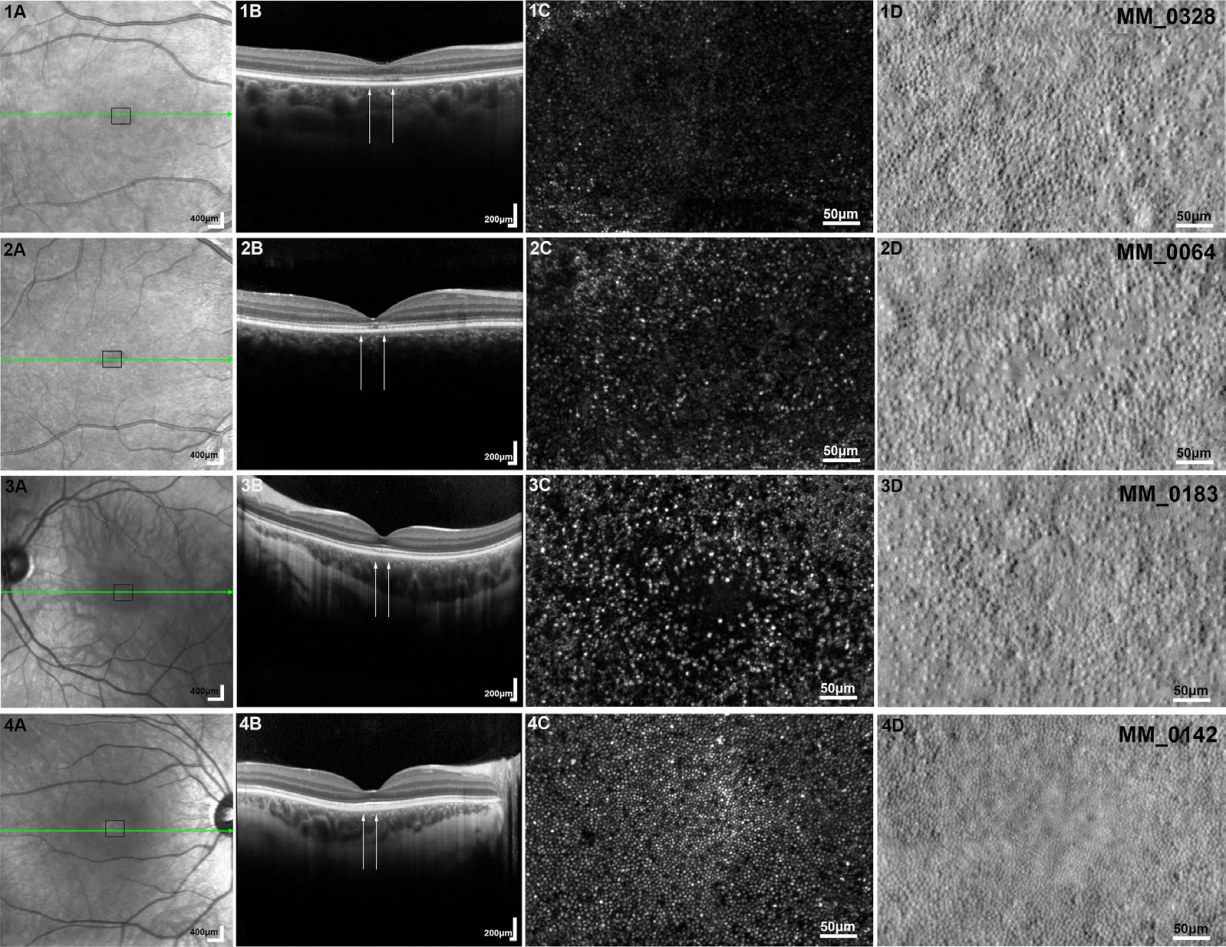
Adaptive optics scanning laser ophthalmoscopy (AOSLO) imaging of the cone dysfunction syndromes. Column (A) shows the infrared reflectance (IR) fundus photographs for each subject (1, 2, 3, 4). The green arrow represents the section in which the optical coherence tomography (OCT) (Spectralis HRA+OCT, Heidelberg Engineering, Heidelberg, Germany) presented in column (B) is taken; the black square represents the 450 μm×300 μm region of interest imaged with AOSLO which is presented in columns (C) and (D). Column (B) shows OCT horizontal scans through the fovea and the white arrows mark the corresponding AOSLO area (450 μm wide). Column (**C**) depicts confocal AOSLO (cAOSLO) and column (D) split detection (SD) AOSLO. Subjects (1) and (2) have achromatopsia associated with *CNGB3* and *CNGA3* gene mutations, respectively. (1C/2C) Dark spaces are observed, due to loss of cone waveguiding properties, which correspond to visible foveal cone inner segments in (1D/2D), respectively, with a substantial difference in cone numerosity between the two subjects. (3) A molecularly confirmed subject with blue cone monochromacy. (3C) Dark foveal centre, with a sparse array of large bright spots, which are believed to be S cones, immediately surrounding it. (3D) Remnant inner segment structure. (4) A molecularly confirmed subject with Bornholm eye disease (LIAVA haplotype). (4C) All cones are resolved in cAOSLO, with a few apparent non-waveguiding cones (dark spaces). (4D) SD-AOSLO does not resolve foveal inner segments due to the better preserved mosaic (smaller cone diameters and tighter packing geometry) compared with the other cone dysfunction syndromes. All AOSLO images were acquired using a custom-built AOSLO housed at University College London/Moorfields Eye Hospital, London. Scale bar=50 μm.

This group of disorders has been reviewed in detail previously^40^ (figure 2).

##### Achromatopsia

Early investigations with cAOSLO identified ‘dark spaces’ in the cone mosaic, increased cone spacing and/or decreased cone density in patients with achromatopsia (ACHM) 16 18 41 42 (figure 2: 1C and 2C). Marked variability in the cone mosaic has been observed across patients; with no significant difference between the two most common genotypes, *CNGA3* and *CNGB3*^42 43^; and the rarer *GNAT2* genotype associated with the relatively least disrupted photoreceptor mosaic.^18 44^ Until the advent of SD-AOSLO, it was unknown if these ‘dark spaces’ harboured non-waveguiding cones or indicated loss of cones—with the presence or absence of cones being directly related to potential rescue with intervention. Simultaneous cAOSLO and SD-AOSLO have allowed the identification of cone inner segment structure in these spaces,^17 24 25 45^ with transformational implications on our understanding of ACHM and participant selection for ongoing *CNGA3-*ACHM and *CNGB3-*ACHM gene therapy trials (figure 2: 1D and 2D). Given the potential disconnect between OCT and AO measures of cone integrity and the ability of AO to directly visualise the target cones for gene replacement, non-confocal SD-AOSLO imaging would be the modality of choice to identify patients most likely to benefit from cone-directed rescue.

In the largest AO ophthalmoscopy ACHM study to date (n=52), significantly decreased peak foveal cone densities and increased spacing, using SD-AOSLO in *CNGB3*-ACHM has been reported.^45^ Interestingly, the peak foveal density ranges were shown to overlap between the previously described OCT grades,^43^ in keeping with the aforementioned disconnect.

Reduced reflectivity in the majority of residual cones in *CNGA3* and *CNGB3* has also been noted, with relative preservation in *GNAT2*. Changes in cone reflectivity could potentially provide a clinical trial outcome metric.^18 42^

Directly relevant to the ongoing debate on whether ACHM is significantly progressive,^40^ based on serial OCT and AOSLO, a longitudinal study of *CNGB3*-ACHM, with follow-up of 6–26 months, showed little or no detectable change in foveal cone structure over time.^24^

##### Blue cone monochromacy

Blue cone monochromacy (BCM) is associated with a range of opsin array genotypes, affecting both L and M cones.^40^ The condition is X-linked, and despite female carriers being asymptomatic, cAOSLO has demonstrated variably reduced cone density, increased spacing and disrupted organisation, with phenotypic variability likely relating to random X-chromosome inactivation.^46^ Affected men have a more severe phenotype, although the degree of cone mosaic disruption is also highly variable and may be partly related to specific genotype group.^47^ The L/M interchange haplotypes have been associated with significantly greater residual parafoveal cone structure, with localised loss of waveguiding cones at the fovea. In contrast, the inactivating Cys203Arg missense mutation genotype group is associated with greater loss of waveguiding L/M cones. cAOSLO images of the cone mosaic typically show a dark foveal centre, with a sparse array of large bright spots, which are believed to be S cones, immediately surrounding it (figure 2C).^47 48^ It is possible to estimate with reasonable confidence the number of L and M cones in the parafovea, as they appear as dark gaps within the rod mosaic.^46^ cAOSLO has demonstrated a reduced number of cones in the parafovea (both reflective S cones and non-reflective L cones and M cones) to that of about 25% of normal, with evidence of even greater loss of cone cells in the locus control region deletion genotype group of BCM. Moreover, SD-AOSLO images have revealed remnant inner segment structure (figure 2B) both at the fovea and the parafovea.^48^ Importantly, however, despite low cone density in BCM, the number is higher than that expected for the S cone submosaic, in keeping with remnant L/M cones.^27 49 50^

Overall, these AOSLO imaging studies have identified significant intersubject variability in cone mosaic integrity and illustrate the importance of cellular imaging in the identification of remnant cones that have the potential to be rescued in planned interventions.

##### Oligocone trichromacy and RGS9/R9AP-associated retinopathy (‘Bradyopsia’)

The cone photoreceptor mosaic in three patients with typical oligocone trichromacy has been investigated, and in keeping with the original disease mechanism hypothesis, a decreased number (‘oligocone’) of otherwise normal appearing foveal cones (thereby permitting ‘trichromacy’) were observed, with absence of visible structure beyond the central fovea.^51^ *RGS9*/*R9AP*-associated retinopathy is clinically indistinguishable from oligocone trichromacy, but can be discerned using non-standard extended electrophysiological assessment or molecular genetic testing. However, unlike in oligocone trichromacy, cAOSLO has revealed a normal cone photoreceptor mosaic in subjects with *RGS9*/*R9AP* retinopathy,^51 52^ which is in agreement with the electroretinography (ERG) findings of normal initial response in dark-adapted flicker ERGs performed with a dim stimulus.^53^ Cellular phenotyping is therefore able to readily differentiate between these two conditions with common clinical features—with an intact photoreceptor mosaic in bradyopsia and disruption in oligocone trichromacy.

##### Bornholm eye disease

Similar to BCM, Bornholm eye disease (BED) is an X-linked cone dysfunction syndrome that is associated with mutations in the L/M gene array.^54–58^ Predominantly due to the heterogeneity in the underlying genotype (predominantly L/M interchange haplotypes), the degree of photoreceptor mosaic disruption in affected men is highly variable, with cone density ranging from near normal to more than 75% reduction^59^ (figure 2: 4C and 4D). However, there is also high variability in the appearance of the cone mosaic within brothers who share the same genotype, likely owing to variations in L:M cone ratio.^59 60^ Cone density has been found to correlate with both axial length and the degree of myopia^60^; however, systematic analysis of the relationship between these factors and the specific underlying L/M opsin variant is lacking, due to small numbers of subjects within each genotype group to date. Additionally, previous investigations employing AOSLO imaging have been cross-sectional, so there is a need for longitudinal studies to track larger genetically confirmed cohorts, both for BED and BCM, to determine natural history and thereby better establish the potential for intervention.

#### Rod dysfunction syndromes

##### Congenital stationary night blindness

Godara *et al* reported retinal structure in three patients with *GRM6-*associated congenital stationary night blindness.^61^ They identified a contiguous cone mosaic and normal cone densities with cAOSLO, in keeping with previous histopathology. They identified photoreceptor mosaic integrity and reported thinning of inner retinal layers on OCT, suggesting a functional defect in retinal neurotransmission, rather than a structural photoreceptor defect.^61^

##### Oguchi disease

Oguchi disease is a very rare form of night blindness having the unusual distinguishing features of the Mizuo-Nakamura phenomenon: diffuse fundus discoloration and return to normal colour after prolonged dark adaption.^62^ To probe the underlying basis of this intriguing phenomenon, the photoreceptor mosaic has been investigated, both in light and dark-adapted conditions, in two molecularly confirmed siblings.^61^ Normal photoreceptor densities were identified; however, rod reflectivity (unlike cone) was shown to increase over time, changing from scotopic to photopic conditions, suggesting that rods are responsible for the unique fundus findings in Oguchi disease.^63^

##### Fundus albipunctatus

Using fluorescence AOSLO and cAOSLO, Song *et al* have reported decreased foveal cone density and increased cone spacing at 10° of eccentricity, despite this predominantly being a rod disorder.^64^ No photoreceptors or RPE cells were visualised within the albipunctate spots. Another study also identified decreased perifoveal cone density and mosaic disruption using cAOSLO in *RDH5*-associated fundus albipunctatus.^65^

### Progressive retinal dystrophies

#### Rod-cone dystrophies

##### Non-syndromic retinitis pigmentosa

In X-linked *RPGR*-associated retinopathy, there is a phenotypic heterogeneity (both intrafamilial and interfamilial) among affected men.^66^ Female carriers’ phenotypes can vary widely, ranging from asymptomatic to severely affected (although not to the extent of affected men) and almost always present with a radial pattern of increased retinal reflectivity, the so-called tapetal-like reflex.^67^ The patchy appearance of rods and cones observed both *ex vivo* and *in vivo* (mosaicism) is believed to be due to random X-chromosome inactivation.

**Figure 3:**
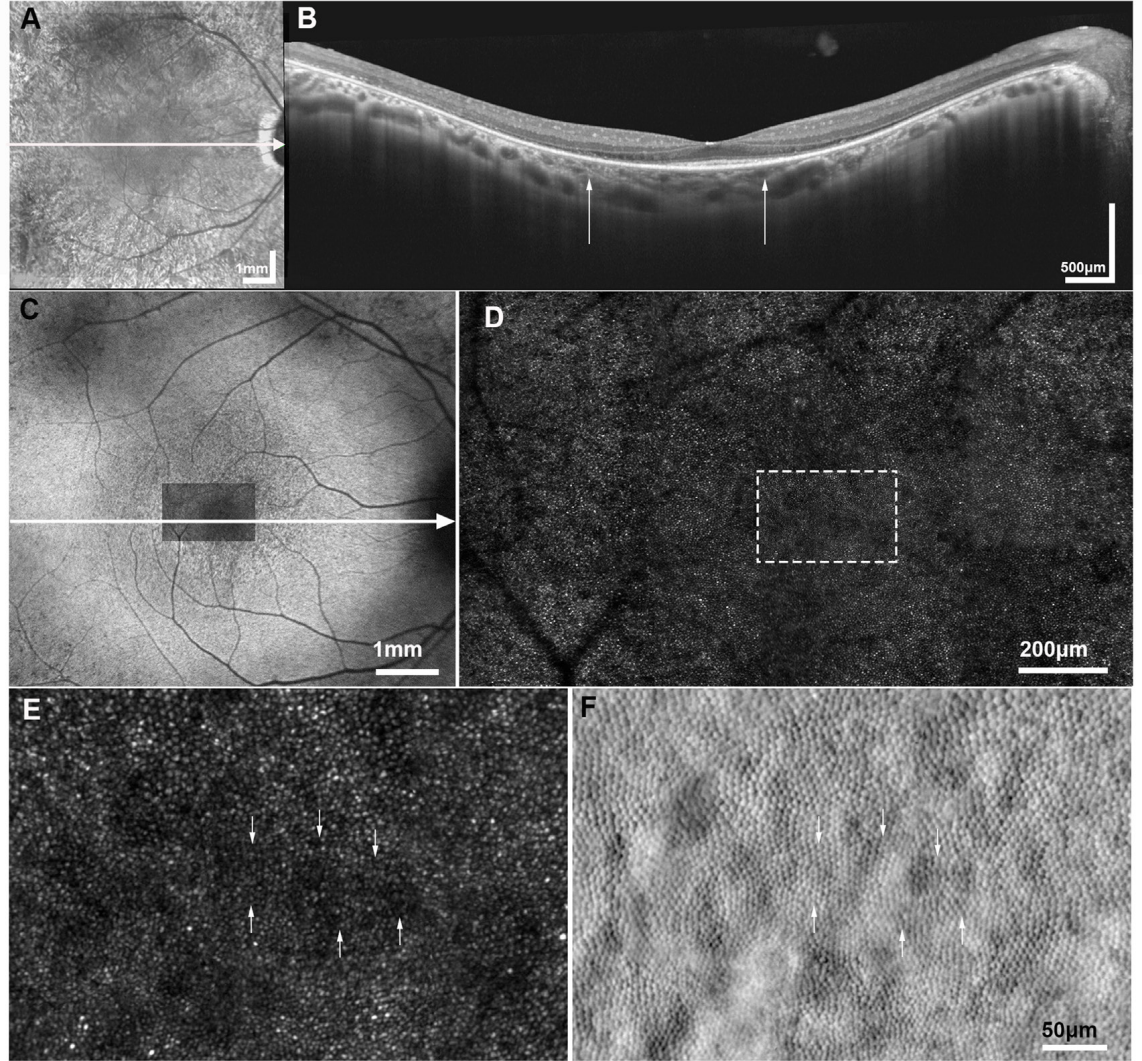
Multimodal imaging of retinitis pigmentosa. (A) Infrared reflectance (IR) fundus photograph of a subject (MM_0205) with X-linked retinitis pigmentosa associated with *RPGR* gene. The white arrow represents the section of the optical coherence tomography (OCT) presented in (B). (B) Horizontal transfoveal OCT line scan, with the white arrows indicating the width of the corresponding AOSLO imaged area in (D). (C) Fundus autofluorescence imaging, with the confocal AOSLO (cAOSLO) imaged area (D) superimposed over the foveal avascular zone and the white arrow represents the section presented in the OCT scan (B). (D) cAOSLO imaging revealing a disrupted waveguiding mosaic, not as uniform in appearance as in a healthy subject (figure 1). (E) Magnification of cAOSLO over the estimated foveal centre (marked with a white dashed square in (D)) shows irregularly waveguiding cones, which appear dim (some are indicated with white arrows); and (F) the corresponding split detection AOSLO in exact spatial registration showing relatively healthy-appearing cone inner segments, the white arrows indicate the corresponding inner segments for the irregularly waveguiding cones identified with white arrows in (E).

Several studies have reported a decrease in cone density and/or increased cone spacing using cAOSLO in patients with retinitis pigmentosa (RP), with approximately half of the subjects having an established genetic diagnosis^68–73^ (figure 3). Sun *et al* 21 examined both patients with RP and Usher syndrome (USH) (see below) using cAOSLO and SD-AOSLO, and found that foveal cone density was reduced by up to 38% before VA was affected, without any visible findings on OCT (however, increased cone spacing was not identified in isolated RP cases). This was in keeping with a previous study reporting normal VA and retinal sensitivity in patients with up to 62% reduction in peak cone density.^73^ These studies illustrate the remarkable redundancy in cone populations, the importance of multimodal imaging and the disconnect between retinal structure and function, with major implications for gene therapy, and also stem cell replacement strategies—including the potential need to successfully integrate smaller numbers of cones than previously believed.

A phase II/III trial has been undertaken with intravitreal implants of encapsulated human RPE cells engineered to continuously secrete ciliary neurotrophic factor (CNTF) protein in patients with early-stage and late-stage RP.^74^ Patients were randomly assigned to receive a high-dose or low-dose implant in one eye and sham surgery in the fellow eye. Primary endpoints were change in BCVA at 12 months for late-stage RP and change in visual field sensitivity at 12 months for early RP. Neither study showed a therapeutic benefit. However, a pilot study using AOSLO in three patients with CNTF implants over a 24-month period found that cone density remained stable in eyes with a CNTF implant, whereas there was continued cone loss in untreated fellow eyes, suggesting that more sensitive metrics are needed as primary outcome measures in progressive diseases such as RP.^71^

##### Usher syndrome

AOSLO has been previously undertaken in one patient with USH type II, and three patients with USH type III.^71 75^ In USH-III, a relative preservation of foveal cone density was observed, with loss of cone structure in areas of absent retinal sensitivity.^75^ Using cAOSLO and SD-AOSLO as complementary modalities, Sun *et al* 21 identified lower foveal and parafoveal cone densities in USH-II (n=4, *USH2A*) compared with non-syndromic RP (n=9 (2 X-linked RP *RPGR*; 3 autosomal recessive RP=2 *USH2A* and 1 *EYS*; 4 autosomal dominant RP=3 *RHO* and 1 *RP1*)) despite the normal appearance of interdigitation (IZ) and ellipsoid zones (EZ) on OCT, which was attributed by the authors to the decreased number of normal waveguiding cones (outer segment defects), possibly a result of the different molecular pathways affected in each condition and the localisation of the affected protein either in the connecting cilium or outer segment.^21^

#### Cone and cone-rod dystrophies

##### Cone-rod dystrophy

Using an AO flood-illuminated ophthalmoscope,^76^ one subject has been imaged showing a lack of waveguiding cones within clinically apparent atrophic regions and a contiguous cone mosaic elsewhere with enlarged cones and reduced peak cone densities. A correlation was observed between cone densities and multifocal ERG (mfERG) peak amplitudes. Similar observations, including structure-function correlation with mfERG, have been observed in three further studies of nine subjects in total.^69 77 78^ AOSLO cone spacing measurements also correlated significantly with mfERG amplitude, retinal sensitivity and VA.^69 76–78^

##### Cone dystrophy with supernormal rod responses

Vincent *et al* assessed macular morphology in cone dystrophy with supernormal rod responses with cAOSLO and reported decreased cone densities and a disrupted mosaic, with cones in groups, surrounded by patches of absent or non-waveguiding cones.^79^ It will be of interest to further investigate subjects with SD-AOSLO to probe whether the non-waveguiding cones are indeed absent or whether inner segments are present.

#### Chorioretinal dystrophies

##### Choroideremia

Patchy cone loss in symptomatic carriers and a normal photoreceptor mosaic in asymptomatic carriers have been observed.^80^ Disrupted parafoveal mosaics, with increased cone spacing, were seen in affected men, with more regular spacing near the borders of atrophy.^80^ In combination with OCT findings, likely simultaneous degeneration of the RPE and photoreceptors was suggested. The largest multimodal study to date including the use of cAOSLO^81^ describes a relatively intact central retina with a normal or reduced cone density at 0.5 mm eccentricity; and an abrupt loss of cones at the border of RPE atrophy, as well as hyper-reflective clumps of cones in younger patients (<30 years) and bubble-like lesions within the choroid; findings also identified by Sun *et al*.^22^ No RPE cells were visible in areas of cone loss, with IZ dropout preceding EZ disruption.^81^ Investigators thereby proposed that choroideremia (CHM) is primarily an RPE disorder followed by photoreceptor degeneration, with implications for intervention and the ongoing debate on cellular pathogenesis of CHM.

Only by using non-confocal SD-AOSLO^22^ has reliable visualisation of cones been possible in the bordering areas of atrophy, with abnormal and heterogeneous morphology, density and diameter. The cone mosaic terminates sharply before those areas, in direct contrast to previously reported RP transition zone imaging, which may relate to the likely primary RPE pathology in CHM. This study also concluded that RPE degeneration precedes photoreceptor loss.

These studies have resulted in AOSLO being incorporated in many ongoing CHM gene therapy trials and natural history studies.^22 80 81^

## Discussion and future possibilities

IRDs are the most genetically and phenotypically heterogeneous conditions in medicine, with certain genotypes being extremely rare making it challenging to establish large informative cohorts, suggesting the need for multicentre studies. Many of the studies incorporated in our review highlight the need for longitudinal monitoring. Insights into disease progression are of great value including identification of the optimal therapeutic window and participant stratification.

AO ophthalmoscopy offers invaluable identification of structural detail on a cellular level, with several studies described herein exploring correlation between structure and function. Evolving AO-guided retinal sensitivity assessments (‘nanoperimerty’) will better allow correlation between cellular imaging and functional testing with exquisite retinotopic precision.^82–84^

One major common limitation is the challenge in imaging patients with nystagmus (eg, ACHM) and/or poor fixation (eg, STGD). Eye tracking systems incorporated into AO systems^85–87^ can allow imaging of more subjects and improve data acquisition. Moreover, image processing and analysis are substantial bottlenecks that developments in, for example, machine learning, will hopefully solve in the near future, thereby allowing a broader application of AO technology.

While our review concentrates on IRDs, AO has been applied to many other conditions including albinism,^14^ age-related macular degeneration,^88 89^ diabetic retinopathy^90^ and autoimmune retinopathy,^91^ and also in basic and applied research, including facilitating insights in visual system neurophysiology.^92^

## Conclusions

AO is a rapidly evolving field, which has a place in diagnosis, advice on prognosis, monitoring and management of IRDs. It can also probe underlying pathophysiology and facilitate improved understanding of cellular retinal anatomy and biology. We anticipate an increasing use of AO systems in the future due to the complementary information they provide compared with other imaging modalities and the ability to target functional measurements on individual cells, with particular application in longitudinal natural history studies and ongoing/planned interventional trials both for participant selection and monitoring treatment safety and efficacy.

## Methods of literature search

PubMed was searched for articles related to AO and IRDs up to June 2017 with key words: Adaptive Optics, AO, AOSLO, Retinal Imaging individually and in combination with the conditions’ name (eg, Achromatopsia) as well as their abbreviations (eg, ACHM).

## References

[1] Liew G, Michaelides M, Bunce C A comparison of the causes of blindness certifications in England and Wales in working age adults (16-64 years), 1999-2000 with 2009-2010. BMJ Open 2014;4:e004015 10.1136/bmjopen-2013-004015 PMC3927710 24525390

[2] Smith J, Ward D, Michaelides M, et al New and emerging technologies for the treatment of inherited retinal diseases: a horizon scanning review. Eye 2015;29:1131–40. 10.1038/eye.2015.115 26113499 PMC4565944

[3] Huang D, Swanson EA, Lin CP, et al Optical coherence tomography. Science 1991;254:1178–81. 10.1126/science.1957169 1957169 PMC4638169

[4] de Boer JF, Leitgeb R, Wojtkowski M Twenty-five years of optical coherence tomography: the paradigm shift in sensitivity and speed provided by Fourier domain OCT [Invited]. Biomed Opt Express 2017;8:3248–80. 10.1364/BOE.8.003248 28717565 PMC5508826

[5] Walsh G, Charman WN, Howland HC Objective technique for the determination of monochromatic aberrations of the human eye. Journal of the Optical Society of America. A, Optics and image science 1984;1:987–92. 6481506 10.1364/josaa.1.000987

[6] Liang J, Williams DR, Miller DT Supernormal vision and high-resolution retinal imaging through adaptive optics. Journal of the Optical Society of America. A, Optics, image science, and vision 1997;14:2884–92. 10.1364/josaa.14.002884 9379246

[7] Roorda A, Williams DR The arrangement of the three cone classes in the living human eye. Nature 1999;397:520–2. 10.1038/17383 10028967

[8] Dubra A, Sulai Y, Norris JL, et al Noninvasive imaging of the human rod photoreceptor mosaic using a confocal adaptive optics scanning ophthalmoscope. Biomed Opt Express 2011;2:1864–76. 10.1364/BOE.2.001864 21750765 PMC3130574

[9] Rossi EA, Chung M, Dubra A, et al Imaging retinal mosaics in the living eye. Eye 2011;25:301–8. 10.1038/eye.2010.221 21390064 PMC3178316

[10] Merino D, Loza-Alvarez P Adaptive optics scanning laser ophthalmoscope imaging: technology update. Clin Ophthalmol 2016;10:743–55. 10.2147/OPTH.S64458 27175057 PMC4854423

[11] Roorda A, Duncan JL Adaptive optics ophthalmoscopy. Annu Rev Vis Sci 2015;1:19–50. 10.1146/annurev-vision-082114-035357 26973867 PMC4786023

[12] Roorda A, Williams DR Optical fiber properties of individual human cones. J Vis 2002;2:4–12. 10.1167/2.5.4 12678654

[13] Zhang T, Godara P, Blanco ER, et al Variability in human cone topography assessed by adaptive optics scanning laser ophthalmoscopy. Am J Ophthalmol 2015;160:290–300. 10.1016/j.ajo.2015.04.034 25935100 PMC4506858

[14] Wilk MA, McAllister JT, Cooper RF, et al Relationship between foveal cone specialization and pit morphology in albinism. Invest Ophthalmol Vis Sci 2014;55:4186–98. 10.1167/iovs.13-13217 24845642 PMC4098060

[15] Cooper RF, Dubis AM, Pavaskar A, et al Spatial and temporal variation of rod photoreceptor reflectance in the human retina. Biomed Opt Express 2011;2:2577–89. 10.1364/BOE.2.002577 21991550 PMC3184867

[16] Merino D, Duncan JL, Tiruveedhula P, et al Observation of cone and rod photoreceptors in normal subjects and patients using a new generation adaptive optics scanning laser ophthalmoscope. Biomed Opt Express 2011;2:2189–201. 10.1364/BOE.2.002189 21833357 PMC3149518

[17] Scoles D, Sulai YN, Langlo CS, et al In vivo imaging of human cone photoreceptor inner segments. Invest Ophthalmol Vis Sci 2014;55:4244–51. 10.1167/iovs.14-14542 24906859 PMC4095721

[18] Dubis AM, Cooper RF, Aboshiha J, et al Genotype-dependent variability in residual cone structure in achromatopsia: toward developing metrics for assessing cone health. Invest Ophthalmol Vis Sci 2014;55:7303–11. 10.1167/iovs.14-14225 25277229 PMC4235328

[19] Razeen MM, Cooper RF, Langlo CS, et al Correlating Photoreceptor Mosaic Structure to Clinical Findings in Stargardt Disease. Transl Vis Sci Technol 2016;5:6 10.1167/tvst.5.2.6 PMC4790429 26981328

[20] Scoles D, Flatter JA, Cooper RF, et al Assessing photoreceptor structure associated with ellipsoid zone disruptions visualized with optical coherence tomography. Retina 2016;36:91–103. 10.1097/IAE.0000000000000618 26166796 PMC4843118

[21] Sun LW, Johnson RD, Langlo CS, et al Assessing Photoreceptor Structure in Retinitis Pigmentosa and Usher Syndrome. Invest Ophthalmol Vis Sci 2016;57:2428–42. 10.1167/iovs.15-18246 27145477 PMC5089122

[22] Sun LW, Johnson RD, Williams V, et al Multimodal Imaging of Photoreceptor Structure in Choroideremia. PLoS One 2016;11:e0167526 10.1371/journal.pone.0167526 27936069 PMC5147929

[23] Scoles D, Sulai YN, Cooper RF, et al Photoreceptor inner segment morphology in best vitelliform macular dystrophy. Retina 2017;37:741–8. 10.1097/IAE.0000000000001203 27467379 PMC5362286

[24] Langlo CS, Erker LR, Parker M, et al Repeatability and longitudinal assessment of foveal cone structure in cngb3-associated achromatopsia. Retina 2017;37:1956–66. 10.1097/IAE.0000000000001434 28145975 PMC5537050

[25] Abozaid MA, Langlo CS, Dubis AM, et al Reliability and repeatability of cone density measurements in patients with congenital achromatopsia. Adv Exp Med Biol 2016;854:277–83. 10.1007/978-3-319-17121-0_37 26427422 PMC4839591

[26] Cooper RF, Sulai YN, Dubis AM, et al Effects of intraframe distortion on measures of cone mosaic geometry from adaptive optics scanning light ophthalmoscopy. Transl Vis Sci Technol 2016;5:10 10.1167/tvst.5.1.10 PMC4771077 26933523

[27] Curcio CA, Sloan KR, Kalina RE, et al Human photoreceptor topography. J Comp Neurol 1990;292:497–523. 10.1002/cne.902920402 2324310

[28] Cooper RF, Wilk MA, Tarima S, et al Evaluating Descriptive Metrics of the Human Cone Mosaic. Invest Ophthalmol Vis Sci 2016;57:2992–3001. 10.1167/iovs.16-19072 27273598 PMC4898203

[29] Garrioch R, Langlo C, Dubis AM, et al Repeatability of in vivo parafoveal cone density and spacing measurements. Optom Vis Sci 2012;89:632–43. 10.1097/OPX.0b013e3182540562 22504330 PMC3348369

[30] Cooper RF, Lombardo M, Carroll J, et al Methods for investigating the local spatial anisotropy and the preferred orientation of cones in adaptive optics retinal images. Vis Neurosci 2016;33:E005 10.1017/S0952523816000018 27484961 PMC5068353

[31] Zhang Y, Rha J, Jonnal R, et al Adaptive optics parallel spectral domain optical coherence tomography for imaging the living retina. Opt Express 2005;13:4792–811. 10.1364/OPEX.13.004792 19495398

[32] Tanna P, Strauss RW, Fujinami K, et al Stargardt disease: clinical features, molecular genetics, animal models and therapeutic options. Br J Ophthalmol 2017;101:25–30. 10.1136/bjophthalmol-2016-308823 27491360 PMC5256119

[33] Chen Y, Ratnam K, Sundquist SM, et al Cone photoreceptor abnormalities correlate with vision loss in patients with Stargardt disease. Invest Ophthalmol Vis Sci 2011;52:3281–92. 10.1167/iovs.10-6538 21296825 PMC3109028

[34] Song H, Rossi EA, Latchney L, et al Cone and rod loss in Stargardt disease revealed by adaptive optics scanning light ophthalmoscopy. JAMA Ophthalmol 2015;133:1198–203. 10.1001/jamaophthalmol.2015.2443 26247787 PMC4600048

[35] Tanna P, Kasilian M, Strauss R, et al Reliability and repeatability of cone density measurements in patients with stargardt disease and RPGR-associated retinopathy. Invest Ophthalmol Vis Sci 2017;58:3608–15. 10.1167/iovs.17-21904 28738413 PMC5525557

[36] Scholl HP, Strauss RW, Singh MS, et al Emerging therapies for inherited retinal degeneration. Sci Transl Med 2016;8:368rv6 10.1126/scitranslmed.aaf2838 27928030

[37] Kong X, Strauss RW, Cideciyan AV, et al Visual acuity change over 12 months in the prospective progression of atrophy secondary to stargardt disease (progstar) study: progstar report number 6. Ophthalmology 2017;124:1640–51. 10.1016/j.ophtha.2017.04.026 28549516

[38] Kay DB, Land ME, Cooper RF, et al Outer retinal structure in best vitelliform macular dystrophy. JAMA Ophthalmol 2013;131:1207–15. 10.1001/jamaophthalmol.2013.387 23765342 PMC3968428

[39] Duncan JL, Ratnam K, Birch DG, et al Abnormal cone structure in foveal schisis cavities in X-linked retinoschisis from mutations in exon 6 of the RS1 gene. Invest Ophthalmol Vis Sci 2011;52:9614–23. 10.1167/iovs.11-8600 22110067 PMC3341122

[40] Aboshiha J, Dubis AM, Carroll J, et al The cone dysfunction syndromes. Br J Ophthalmol 2016;100:115–21. 10.1136/bjophthalmol-2014-306505 25770143 PMC4717370

[41] Carroll J, Choi SS, Williams DR In vivo imaging of the photoreceptor mosaic of a rod monochromat. Vision Res 2008;48:2564–8. 10.1016/j.visres.2008.04.006 18499214 PMC2582057

[42] Genead MA, Fishman GA, Rha J, et al Photoreceptor structure and function in patients with congenital achromatopsia. Invest Ophthalmol Vis Sci 2011;52:7298–308. 10.1167/iovs.11-7762 21778272 PMC3183969

[43] Sundaram V, Wilde C, Aboshiha J, et al Retinal structure and function in achromatopsia: implications for gene therapy. Ophthalmology 2014;121:234–45. 10.1016/j.ophtha.2013.08.017 24148654 PMC3895408

[44] Ueno S, Nakanishi A, Kominami T, et al In vivo imaging of a cone mosaic in a patient with achromatopsia associated with a GNAT2 variant. Jpn J Ophthalmol 2017;61:92–8. 10.1007/s10384-016-0484-7 27718025

[45] Langlo CS, Patterson EJ, Higgins BP, et al Residual Foveal Cone Structure in CNGB3-Associated Achromatopsia. Invest Ophthalmol Vis Sci 2016;57:3984–95. 10.1167/iovs.16-19313 27479814 PMC4978151

[46] Carroll J, Rossi EA, Porter J, et al Deletion of the X-linked opsin gene array locus control region (LCR) results in disruption of the cone mosaic. Vision Res 2010;50:1989–99. 10.1016/j.visres.2010.07.009 20638402 PMC3005209

[47] Carroll J, Dubra A, Gardner JC, et al The effect of cone opsin mutations on retinal structure and the integrity of the photoreceptor mosaic. Invest Ophthalmol Vis Sci 2012;53:8006–15. 10.1167/iovs.12-11087 23139274 PMC3816954

[48] Patterson EJ, Kasilian M, Kalitzeos A, et al “Assessing cone photoreceptor structure in patients with mutations in the OPN1LW/OPN1MW gene array”. Invest. Ophthalmol. Vis. Sci 2017:E-Abstract 1257.

[49] Cideciyan AV, Hufnagel RB, Carroll J, et al Human cone visual pigment deletions spare sufficient photoreceptors to warrant gene therapy. Hum Gene Ther 2013;24:993–1006. 10.1089/hum.2013.153 24067079 PMC3868405

[50] Curcio CA, Allen KA, Sloan KR, et al Distribution and morphology of human cone photoreceptors stained with anti-blue opsin. J Comp Neurol 1991;312:610–24. 10.1002/cne.903120411 1722224

[51] Michaelides M, Rha J, Dees EW, et al Integrity of the cone photoreceptor mosaic in oligocone trichromacy. Invest Ophthalmol Vis Sci 2011;52:4757–64. 10.1167/iovs.10-6659 21436275 PMC3175968

[52] Strauss RW, Dubis AM, Cooper RF, et al Retinal architecture in ​RGS9- and ​R9AP-associated retinal dysfunction (bradyopsia). Am J Ophthalmol 2015;160:1269–75. 10.1016/j.ajo.2015.08.032 26343007 PMC4653116

[53] Michaelides M, Li Z, Rana NA, et al Novel mutations and electrophysiologic findings in RGS9- and R9AP-associated retinal dysfunction (Bradyopsia). Ophthalmology 2010;117:120–7. 10.1016/j.ophtha.2009.06.011 19818506

[54] Schwartz M, Haim M, Skarsholm D X-linked myopia: bornholm eye disease. linkage to DNA markers on the distal part of Xq. Clin Genet 1990;38:281–6. 1980096

[55] Young TL, Deeb SS, Ronan SM, et al X-linked high myopia associated with cone dysfunction. Arch Ophthalmol 2004;122:897–908. 10.1001/archopht.122.6.897 15197065

[56] Michaelides M, Johnson S, Bradshaw K, et al X-linked cone dysfunction syndrome with myopia and protanopia. Ophthalmology 2005;112:1448–54. 10.1016/j.ophtha.2005.02.021 15953640

[57] Guo X, Xiao X, Li S, et al Nonsyndromic high myopia in a Chinese family mapped to MYP1: linkage confirmation and phenotypic characterization. Arch ophthalmol 2010;128:1473–9. 21060050 10.1001/archophthalmol.2010.270

[58] Li J, Gao B, Guan L, et al Unique variants in OPN1LW cause both syndromic and nonsyndromic x-linked high myopia mapped to MYP1. Invest Ophthalmol Vis Sci 2015;56:4150–5. 10.1167/iovs.14-16356 26114493

[59] Neitz J, Wagner-Schuman M, Dubra A, et al Cone mosaic disruption caused by L/M opsin mutations in bornholm eye disease. Invest Ophthalmol Vis Sci 2011;52:4896–96.

[60] Patterson EJ, Wilk M, Langlo CS, et al Cone photoreceptor structure in patients with x-linked cone dysfunction and red-green color vision deficiency. Invest Ophthalmol Vis Sci 2016;57:3853–63. 10.1167/iovs.16-19608 27447086 PMC4968428

[61] Godara P, Cooper RF, Sergouniotis PI, et al Assessing retinal structure in complete congenital stationary night blindness and Oguchi disease. Am J Ophthalmol 2012;154:987–1001. 10.1016/j.ajo.2012.06.003 22959359 PMC3498541

[62] Dryja TP Molecular genetics of Oguchi disease, fundus albipunctatus, and other forms of stationary night blindness: LVII Edward Jackson Memorial Lecture. Am J Ophthalmol 2000;130:547–63. 10.1016/S0002-9394(00)00737-6 11078833

[63] Weiss ER, Ducceschi MH, Horner TJ, et al Species-specific differences in expression of G-protein-coupled receptor kinase (GRK) 7 and GRK1 in mammalian cone photoreceptor cells: implications for cone cell phototransduction. J Neurosci 2001;21:9175–84. 11717351 10.1523/JNEUROSCI.21-23-09175.2001 PMC6763890

[64] Song H, Latchney L, Williams D, et al Fluorescence adaptive optics scanning laser ophthalmoscope for detection of reduced cones and hypoautofluorescent spots in fundus albipunctatus. JAMA Ophthalmol 2014;132:1099–104. 10.1001/jamaophthalmol.2014.1079 24922193 PMC4162840

[65] Makiyama Y, Ooto S, Hangai M, et al Cone abnormalities in fundus albipunctatus associated with RDH5 mutations assessed using adaptive optics scanning laser ophthalmoscopy. Am J Ophthalmol 2014;157:e1-4:558–70. 10.1016/j.ajo.2013.10.021 24246574

[66] Tee JJ, Smith AJ, Hardcastle AJ, et al RPGR-associated retinopathy: clinical features, molecular genetics, animal models and therapeutic options. Br J Ophthalmol 2016;100:1022–7. 10.1136/bjophthalmol-2015-307698 26843488

[67] Fishman GA, Weinberg AB, McMahon TT X-linked recessive retinitis pigmentosa. clinical characteristics of carriers. Arch Ophthalmol 1986;104:1329–35. 3753283 10.1001/archopht.1986.01050210083030

[68] Makiyama Y, Ooto S, Hangai M, et al Macular cone abnormalities in retinitis pigmentosa with preserved central vision using adaptive optics scanning laser ophthalmoscopy. PLoS One 2013;8:e79447 10.1371/journal.pone.0079447 24260224 PMC3834127

[69] Duncan JL, Zhang Y, Gandhi J, et al High-resolution imaging with adaptive optics in patients with inherited retinal degeneration. Invest Ophthalmol Vis Sci 2007;48:3283–91. 10.1167/iovs.06-1422 17591900

[70] Park SP, Lee W, Bae EJ, et al Early structural anomalies observed by high-resolution imaging in two related cases of autosomal-dominant retinitis pigmentosa. Ophthalmic Surg Lasers Imaging Retina 2014;45:469–73. 10.3928/23258160-20140908-01 25215869 PMC4377129

[71] Talcott KE, Ratnam K, Sundquist SM, et al Longitudinal study of cone photoreceptors during retinal degeneration and in response to ciliary neurotrophic factor treatment. Invest Ophthalmol Vis Sci 2011;52:2219–26. 10.1167/iovs.10-6479 21087953 PMC3080173

[72] Zayit-Soudry S, Sippl-Swezey N, Porco TC, et al Repeatability of cone spacing measures in eyes with inherited retinal degenerations. Invest Ophthalmol Vis Sci 2015;56:6179–89. 10.1167/iovs.15-17010 26416092

[73] Ratnam K, Carroll J, Porco TC, et al Relationship between foveal cone structure and clinical measures of visual function in patients with inherited retinal degenerations. Invest Ophthalmol Vis Sci 2013;54:5836–47. 10.1167/iovs.13-12557 23908179 PMC3757906

[74] Birch DG, Weleber RG, Duncan JL, et al Randomized trial of ciliary neurotrophic factor delivered by encapsulated cell intraocular implants for retinitis pigmentosa. Am J Ophthalmol 2013;156:283–92. 10.1016/j.ajo.2013.03.021 23668681 PMC4111936

[75] Ratnam K, Västinsalo H, Roorda A, et al Cone structure in patients with usher syndrome type III and mutations in the Clarin 1 gene. JAMA Ophthalmol 2013;131:67–74. 10.1001/2013.jamaophthalmol.2 22964989 PMC4482614

[76] Wolfing JI, Chung M, Carroll J, et al High-resolution retinal imaging of cone-rod dystrophy. Ophthalmology 2006;113:e1:1014–9. 10.1016/j.ophtha.2006.01.056 16650474

[77] Choi SS, Doble N, Hardy JL, et al In vivo imaging of the photoreceptor mosaic in retinal dystrophies and correlations with visual function. Invest Ophthalmol Vis Sci 2006;47:2080–92. 10.1167/iovs.05-0997 16639019 PMC2583223

[78] Roorda A, Zhang Y, Duncan JL High-resolution in vivo imaging of the RPE mosaic in eyes with retinal disease. Invest Ophthalmol Vis Sci 2007;48:2297–303. 10.1167/iovs.06-1450 17460294

[79] Vincent A, Wright T, Garcia-Sanchez Y, et al Phenotypic characteristics including in vivo cone photoreceptor mosaic in KCNV2-related "cone dystrophy with supernormal rod electroretinogram". Invest Ophthalmol Vis Sci 2013;54:898–908. 10.1167/iovs.12-10971 23221069 PMC3880354

[80] Syed R, Sundquist SM, Ratnam K, et al High-resolution images of retinal structure in patients with choroideremia. Invest Ophthalmol Vis Sci 2013;54:950–61. 10.1167/iovs.12-10707 23299470 PMC3564452

[81] Morgan JI, Han G, Klinman E, et al High-resolution adaptive optics retinal imaging of cellular structure in choroideremia. Invest Ophthalmol Vis Sci 2014;55:6381–97. 10.1167/iovs.13-13454 25190651 PMC4193760

[82] Tuten WS, Tiruveedhula P, Roorda A Adaptive optics scanning laser ophthalmoscope-based microperimetry. Optom Vis Sci 2012;89:563–74. 10.1097/OPX.0b013e3182512b98 22446720 PMC3348404

[83] Bruce KS, Harmening WM, Langston BR, et al Normal perceptual sensitivity arising from weakly reflective cone photoreceptors. Invest Ophthalmol Vis Sci 2015;56:4431–8. 10.1167/iovs.15-16547 26193919 PMC4509056

[84] Wang Q, Tuten WS, Lujan BJ, et al Adaptive optics microperimetry and OCT images show preserved function and recovery of cone visibility in macular telangiectasia type 2 retinal lesions. Invest Ophthalmol Vis Sci 2015;56:778–86. 10.1167/iovs.14-15576 25587056 PMC4315435

[85] Sheehy CK, Tiruveedhula P, Sabesan R, et al Active eye-tracking for an adaptive optics scanning laser ophthalmoscope. Biomed Opt Express 2015;6:2412–23. 10.1364/BOE.6.002412 26203370 PMC4505698

[86] Privitera CM, Sabesan R, Winter S, et al Eye-tracking technology for real-time monitoring of transverse chromatic aberration. Opt Lett 2016;41:1728–31. 10.1364/OL.41.001728 27082330 PMC5322945

[87] Tam J, Roorda A Speed quantification and tracking of moving objects in adaptive optics scanning laser ophthalmoscopy. J Biomed Opt 2011;16:036002 10.1117/1.3548880 21456866 PMC3081139

[88] Gocho K, Sarda V, Falah S, et al Adaptive optics imaging of geographic atrophy. Invest Ophthalmol Vis Sci 2013;54:3673–80. 10.1167/iovs.12-10672 23620431

[89] Querques G, Kamami-Levy C, Georges A, et al Adaptive optics imaging of foveal sparing in geographic atrophy secondary to age-related macular degeneration. Retina 2016;36:247–54. 10.1097/IAE.0000000000000692 26200512

[90] Burns SA, Elsner AE, Chui TY, et al In vivo adaptive optics microvascular imaging in diabetic patients without clinically severe diabetic retinopathy. Biomed Opt Express 2014;5:961–74. 10.1364/BOE.5.000961 24688827 PMC3959854

[91] Davoudi S, Ebrahimiadib N, Yasa C, et al Outcomes in autoimmune retinopathy patients treated with rituximab. Am J Ophthalmol 2017;180:124–32. 10.1016/j.ajo.2017.04.019 28483493

[92] Marcos S, Werner JS, Burns SA, et al Vision science and adaptive optics, the state of the field. Vision Res 2017;132:3–33. 10.1016/j.visres.2017.01.006 28212982 PMC5437977

