# Supplementary material for: Adaptive optics imaging of inherited retinal diseases
Source: Br J Ophthalmol. 2017 Nov 15;102(8):1028–35. doi: 10.1136/bjophthalmol-2017-311328 (PMC6059037; doi:10.1136/bjophthalmol-2017-311328)
Supplement: Supplementary file 1 [file bjophthalmol-2017-311328supp001.pdf]

Supplementary Table 1

AUTHOR	YEAR	AO DETAILS	Subjects (N=)
**Macular Dystrophies**			
Stargardt Disease			
Chen, Ratnam et al.	2011	cAOSLO	12
Song, Rossi et al.	2015	cAOSLO	2
Razeen, Cooper et al.	2016	cAOSLO / SD-AOSLO	14
Tanna et al.	2017	cAOSLO / SD-AOSLO	12
Best Disease (BD)			
Kay, Land et al.	2013	cAOSLO	2
Scoles, Sulai et al.	2017	cAOSLO / SD-AOSLO	2
X-Linked Retinoschisis (XLRS)			
Duncan, Ratnam et al.	2011	cAOSLO	2
**Stationary Dysfunction Syndromes**			
**Cone Dysfunction Syndromes**			
Achromatopsia (ACHM)			
Carroll, Choi et al.	2008	AO-Flood Illuminated Camera	2
Genead, Fishman et al.	2011	AO-Flood Illuminated Camera	12
Merino, Duncan et al.	2011	cAOSLO	1
Dubis, Cooper et al.	2014	cAOSLO	11
Scoles, Sulai et al.	2014	cAOSLO / SD-AOSLO	4
Abozaid, Langlo et al.	2016	cAOSLO / SD-AOSLO	7
Langlo, Patterson et al.	2016	cAOSLO / SD-AOSLO	51
Ueno, Nakanishi et al.	2017	AO-Flood Illuminated Camera	1
Langlo, Erker et al.	2017	cAOSLO / SD-AOSLO	41
Blue Cone Monochromacy (BCM)			
Carroll, Rossi et al.	2010	cAOSLO	4
Carroll, Dubra et al.	2012	cAOSLO	11
Cideciyan, Hufnagel et al.	2013	cAOSLO	20
Oligocone Trichromacy (OT) and RGS9/R9AP-associated Retinopathy ('Bradyopsia')			
Michaelides, Rha et al.	2011	cAOSLO	4
Strauss, Dubis et al.	2015	cAOSLO	3
Patterson, Wilk et al.	2016	cAOSLO	17
**Rod Dysfunction Syndromes**			
Fundus Albipunctatus (FA)			
Song, Latchney et al.	2014	cAOSLO/FAOSLO	1
Makiyama, Ooto et al.	2014	cAOSLO	10
Oguchi Disease and Congenital Stationary Night Blindness (CSNB)			
Godara, Cooper et al.	2012	cAOSLO	3
**Progressive Retinal Dystrophies**			
**Rod-Cone Dystrophies**			
Non-Syndromic Retinitis Pigmentosa (RP) and Usher Syndrome (USH)			
Duncan, Zhang et al.	2007	cAOSLO	5
Talcott, Ratnam et al.	2011	cAOSLO	3 (2 RP+1 USH)
Makiyama, Ooto et al.	2013	cAOSLO	14
Ratnam, Carroll et al.	2013	cAOSLO	26
Park, Lee et al.	2014	cAOSLO	2
Zayit-Soudry, Sippl-Swezey et al.	2015	cAOSLO	20
Sun, Johnson et al.	2016	cAOSLO / SD-AOSLO	11 (7 RP+4 USH)
**Cone and Cone-Rod Dystrophies**			
Cone-Rod Dystrophy (CORD)			
Choi, Doble et al.	2006	AO-Flood Illuminated Camera	3
Wolfing, Chung et al.	2006	AO-Flood Illuminated Camera	1
Duncan, Zhang et al.	2007	cAOSLO	3
Cone Dystrophy with Supernormal Rod Responses (CDSR)			
Vincent, Wright et al.	2013	cAOSLO	7
**Chorioretinal Dystrophies**			
Choroideremia (CHM)			
Syed, Sundquist et al.	2013	cAOSLO	5 (+6 Carriers)
Morgan, Han et al.	2014	cAOSLO	57 (+18 Carriers)
Sun, Johnson et al.	2016	cAOSLO / SD-AOSLO	12
